# Evaluating the Performance of Functionalized Carbon Structures with Integrated Optical Fiber Sensors under Practical Conditions

**DOI:** 10.3390/s18113923

**Published:** 2018-11-14

**Authors:** Kort Bremer, Lourdes S. M. Alwis, Frank Weigand, Michael Kuhne, Yulong Zheng, Marco Krüger, Reinhard Helbig, Bernhard Roth

**Affiliations:** 1 Hannover Centre for Optical Technologies (HOT), Leibniz University Hannover, 30167 Hannover, Germany; Zheng.Yulong@slm-solutions.com (Y.Z.); Bernhard.Roth@hot.uni-hannover.de (B.R.); 2 School of Engineering and the Built Environment, Edinburgh Napier University, Edinburgh EH11 4BN, UK; L.Alwis@napier.ac.uk; 3 Saxon Textile Research Institute (STFI), 09125 Chemnitz, Germany; Frank.Weigand@stfi.de (F.W.); Reinhard.Helbig@stfi.de (R.H.); 4 Materialforschungs-und-Prüfanstalt an der Bauhaus-Universität Weimar (MFPA), 99423 Weimar, Germany; Michael.Kuhne@mfpa.de (M.K.); Marco.Krueger@mfpa.de (M.K.)

**Keywords:** carbon concrete composite, structural health monitoring, optical frequency domain reflectometry, fibre Bragg grating, strain measurement

## Abstract

An Optical Frequency Domain Reflectometry (OFDR) based fiber optic sensor scheme “embedded” in concrete for the purpose of structural health monitoring (SHM) of carbon concrete composites (C^3^) is presented. The design, while strengthening the concrete structure, also aims to monitor common SHM parameters such as strain and cracks. This was achieved by weaving the carbon fiber together with optical fiber, based on a specialized technique that uses an embroidery setup where both the carbon and optical fiber are woven on a water dissolvable polymer substrate. The performance of the sensing scheme was characterized in-situ utilizing the OFDR based technique and the results presented. The sensors embedded on a custom made concrete block were subjected to varying strain via a three point bending test to destruction and the results discussed. The intended dual-achievement of the scheme thus proposed in SHM and strengthening the C^3^ is demonstrated. The suitability of the OFDR scheme for C^3^ is combined with a fibre Bragg grating (FBG)-based approach, and discussed in detail.

## 1. Introduction

The project Carbon Concrete Composite (C^3^) is one of the largest research programmes in the field of building constructions in Germany at present, aiming to replace traditional steel reinforced concrete (SRC) as building material by carbon reinforced concrete (CRC). This arises from the fact that CRC possesses a considerable amount of advantages over SRC, such as light weight, longer lifespan, flexibility (easier to mould), efficiency and immunity to risk of corrosion [1]. In addition to these, its thermal and electrical conductivity properties, together with its ability to be constructed in thin layers with high tensile strength render it an ideal candidate for intelligent building construction.

The use of fiber optic sensors (FOSs) for Structural Health Monitoring (SHM) of reinforced concrete structures is state of the art and within this domain, FOSs are utilized mainly to monitor corrosion of the reinforcing steel and to measure the deformation of the reinforced concrete. For example, fiber optic strain sensors [2], moisture sensors [3] and pH sensors [4] have been developed to monitor the corrosion of rebar. For deformation measurement, FOSs are applied both on the surface of reinforced concrete [5,6] as well as directly embedded in the cement stone matrix [7]. FOSs have also been investigated for aerospace applications, for example, for condition monitoring of carbon fiber reinforced plastic (CFRP) components [8]. Within the context of this particular application, such sensors were developed especially for the detection of cracks [9,10] or delamination [11] of the CFRP.

The rapid uptake of carbon fiber reinforcement by Civil engineering creates a demand, but more of an opportunity, for smart sensors to be embedded in the structure itself for real-time measurement of the structural integrity. The direct integration of FOSs into carbon fiber structure for monitoring the condition of carbon fiber concrete, therefore, represents a new field of research. Compared to CFRP applications, when integrating FOSs in carbon fiber reinforced concrete structures, the highly alkaline environment in concrete and the concrete mechanical stress places special demands on the optical fiber as well as on the bond between optical fiber and carbon fiber reinforcement. Also, the optical glass fiber (OGF) can be destroyed and/or cracking can be experienced by concrete chemical stress induced micro defects on the surface of glass fiber resulting from manufacturing defects or stress corrosion. Thus, the mechanical properties of the OGF must be carefully evaluated in this context. Furthermore, the sensor region can be detached from the carbon reinforcement as a result of mechanical stress, or the composite can be slowly decomposed as a result of the highly alkaline environment in concrete. Thus, in order to ensure functionality across the C^3^ building cycle, both a new embroidery integration process for fabrication and the property of the bond between suitable OGF and carbon fiber reinforcement structures need to be investigated.

Previous work by the authors [12] has focused on OFSs embedded in functionalised carbon structures (FCSs) and textile net structures (TNSs) based on alkaline resistant (AR) glass for SHM of concrete structures. The design was utilized to monitor strain and cracks while at the same time acting as a structural strengthening mechanism. The sensor performance was characterized using Mach-Zehnder Interferometric (MZI) and optical time domain reflectometry (OTDR) techniques, respectively. The work presented here builds up from the aforementioned work [12] and extends further to embed FCSs in concrete blocks and evaluate the performance of the fibre optic sensors. The evaluation of the fibre optic sensors includes FBGs and OFDR. FBGs have the advantages of providing strain measurements with high sensitivity and fast response time compared to OFDR. However, FBGs could detect strain only at specified locations along the fiber while OFDR could be applied for distributed sensing where strain measurement along the entire length of the fiber could be achieved. Therefore, by using FBGs, the sensor performance in terms of sensitivity and hysteresis is characterized, whereas the OFDR method is applied to evaluate the strain profile of concrete. Further theoretical background on FBG and OFDR techniques could be found in the literature [13,14].

## 2. Materials and Methods

### 2.1. Fabrication of FCS

**Figure 1:**
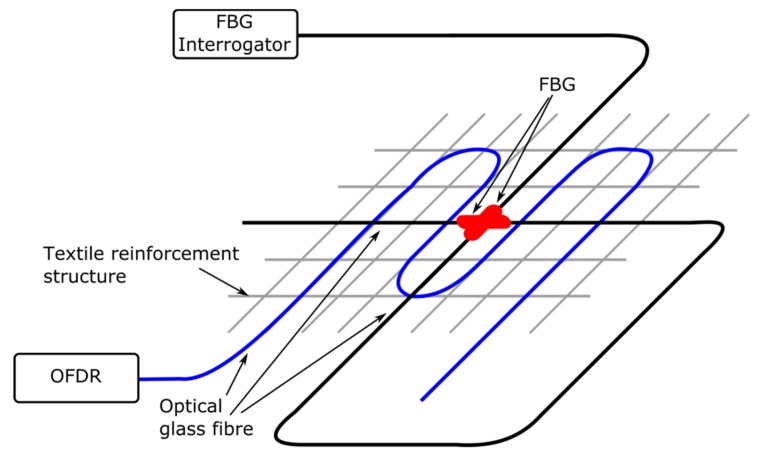
Sketch of the FCS samples for testing under practical conditions.

**Figure 2:**
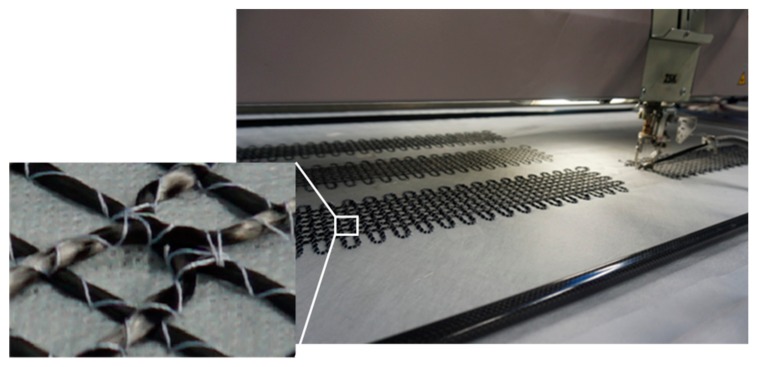
Embroidery machine that was used to fabricate FCSs. The machine was modified to enable simultaneous processing of carbon filaments and optical fibers to a grid-like structure on PVA nonwoven substrates.

The FCS, as illustrated schematically in Figure 1, have been fabricated using an embroidery technique that was developed at the STFI [12]. The developed technique processes carbon filaments and OGFs simultaneously to produce grid-like structures. To achieve this, the carbon filaments and the OGFs are embroidered on a polyvinyl alcohol (PVA) nonwoven substrate. In addition, to obtain the required tailored grid-like structure, several layers of carbon filaments were incorporated subsequently on the PVA nonwoven substrate. Upon completion of the embroidery fabrication process, the nonwoven substrate is then removed by dissolving the PVA in hot water (approx. 50 °C) and only a grid-like structure remains that consists of carbon filaments and OGFs. The advantage of this technique is the possibility of the fabrication of tailored carbon structures, i.e., several layers of carbon filaments with up to 50 k filament fibers (corresponding to 3200 tex), as well as grid structures that are different in shape, depending on the targeted application and purpose. The embroidery of carbon filaments and OGFs on the PVA nonwoven substrate are shown in Figure 2.

In order to investigate the performance of the FOSs, FCSs with 1600 tex and dimension of 500 × 110 mm^2^ have been fabricated using the embroidery technique reported above. Furthermore, the measurement of the spatial strain profile along the OGF was achieved based on an OFDR technique using Corning SMF28E XB fiber with acrylate coating and two Fibercore SM1500 fibres (with FBG sensors and acrylate coating) that had been integrated in the FCS, which is illustrated in Figure 1. The FBGs have been inscribed in-house in photosensitive Fibercore SM1500 fiber using a KrF excimer laser and the phase mask technique [15]. The length of the fabricated FBGs was 7 mm and the Full Width Half Maximum (FWHM) of the resulting spectral reflection peaks was 0.53 nm. Moreover, the fibers have been recoated after the FBG inscription process using UV curable acrylate glue.

### 2.2. Fabrication of Concrete Blocks Containing FCS

**Figure 3:**
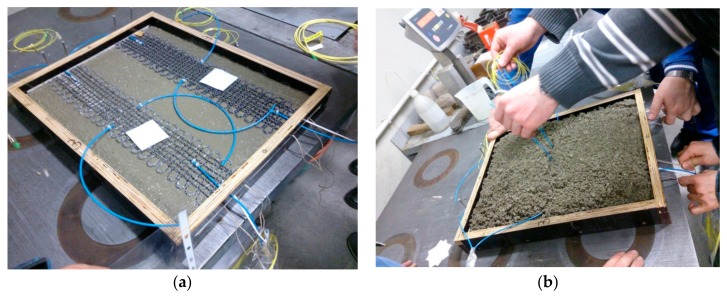
Embedding FCS in concrete; (**a**) Two FCSs with dimensions of 500 × 110 mm^2^ were fixed in a 500 × 400 × 40 mm^3^ mould; (**b**) Plastic tubes applied to protect fiber from breackage at the FCS/concrete interface. The FBGs are positioned at the centre of the FCS.

Upon completion of the fabrication, the FCSs were embedded in concrete blocks in order to evaluate their performance. To do so, first, two FCS samples (500 × 110 mm^2^) were placed in a mould with dimensions of 500 × 400 × 40 mm^3^, which was half filled with liquid concrete. After the FCS samples were fixed at the correct position, the mould was then completely filled with concrete. Following this, the liquid concrete blocks were shaken for five minutes to obtain an even distribution and the concrete was left to set for at least 28 days. After the concrete was complety set, the concrete blocks were seperated into two blocks, where each block incorporated a FCS sample. Furthermore, to protect the optical fiber in the FCS from breackage when integrating the FCS sample into the concrete, the optical fiber was protected at the FCS/concrete interface using plastic tubes and FC/PC pigtails were spliced to the endfaces of the fibers of the FCSs. The FCS sample before (a) and during embedding (b) is illustrated in Figure 3.

In addition, the optical parameters of the FOSs in concrete were recorded and the attenuation of the OGFs embedded in the carbon reinforcement, before and after applying concrete, was determined with the aid of a FiboTec power meter (dB-meter). A summary of the thus determined values of three exemplary samples is presented in Table 1. In addition, none of the OGFs was destroyed and insignificant light attenuation was detected upon integrating the FCS in concrete. The variation of transmitted power before and after applying concrete could be due to the connectors. In summary, the OGFs were not destroyed during integration into concrete in all samples.

### 2.3. Experimental Set-Up to Evaluate Sensor Performance

**Figure 4:**
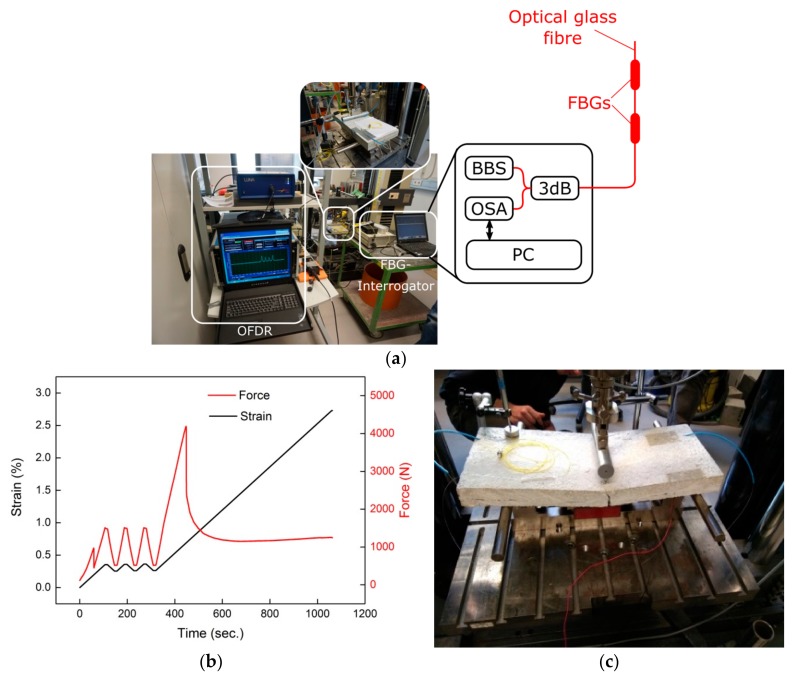
Experimental set-up to evaluate FCSs under practical conditions; (**a**) Concrete block containing FCS connected to the OFDR interrogator and FBG spectrometer; (**b**) Example of strain and force applied to a concrete block under test; (**c**) Concrete block with embedded FCS at the point of break.

The performance of the integrated OFSs was evaluated using a 3-point bending test rig, as illustrated in Figure 4a. While bending the concrete block, strain was introduced in the FCS, and thus in the corresponding OGFs, and therefore the strain transfer from the concrete to the OGFs could be characterized. For this purpose, the concrete specimens were successively placed in the bending test machine and the FOSs were connected to the corresponding interrogators, i.e., an FBG spectrometer and an OFDR interrogator. For the OFDR interrogation, Luna ODiSI-B that uses the software “Optical Distributed Sensor Interrogator B Series” was applied for online monitoring of the spatial strain profile with a spatial strain resolution of 1.28 mm (obtained from Luna ODiSI-B datasheet). The FBG spectrometer setup that measures the reflected light from each sensor utilized a broadband light source (BBS, Opto-Link ASE), a 3 dB coupler (3 dB) as well as an optical spectrum analyser (OSA, Ibsen I-Mon E). Both FBG sensors of the FCS were connected in series and the shift of the Bragg wavelength has been determined using the Ibsen I-Mon E software.

In order to test the concrete specimens under practical conditions, three bending cycles (between 500 and 1500 N) were first carried out with the help of the 3-point bending test rig. The three bending cycles at the beginning of the test procedure were performed in order to specify the hysteresis of the FOSs embedded in the FCS. Following the three bending cycles, the concrete specimens were tested until failure, i.e., breakage. An example of the strain and force applied to a concrete specimen as a function of time using the 3-point bending test is presented in Figure 4b. The fracture of the concrete specimen is indicated by the abrupt force penetration approximately after 400 s in the example shown in Figure 4b. The fracture of a concrete specimen after completing the 3-point bending test is illustrated in Figure 4c. The concrete specimen shows a clear breakage, but the FCS including the OGF was still intact and the OFS is thus capable in determining the measurand even after the breakage of the concrete specimen.

## 3. Results and Discussion

### 3.1. Performance of FBG Sensors

**Figure 5:**
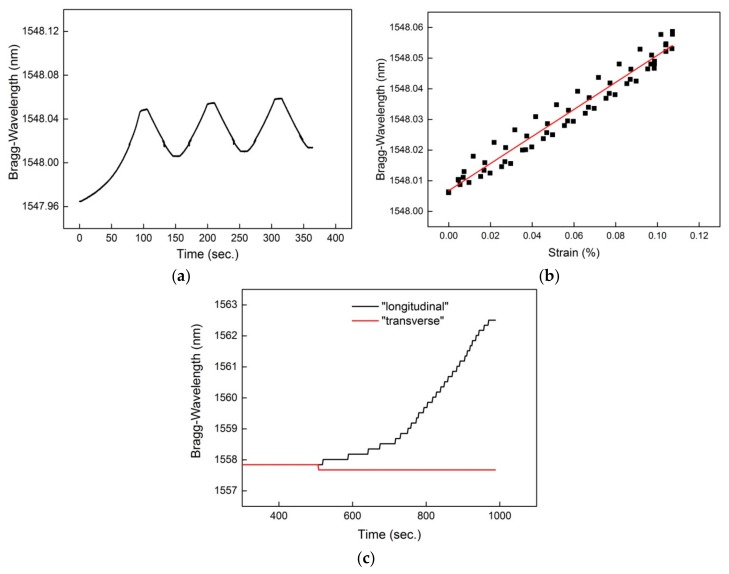
Readings of the FBG sensor of the FCS; (**a**) Sensor response by the FBG that is positioned longitudinal to the load; (**b**) FBG sensor indicates a linear response to applied load; (**c**) Minimal or no sensitivity of the FBG sensors to lateral force.

The results of FBG sensor measurements from the FCS are shown in Figure 5. An example of the sensor readings of the FBG positioned longitudinal to the applied force is illustrated in Figure 5a,b. As can be seen, the FBG sensor was able to detect the three consecutive strain cycles (Figure 5a) with a sensitivity of 0.44 nm/% as well as a relatively low hysteresis (0.011%) (Figure 5b). The sensitivity of two perpendicularly placed FBG sensors to longitudinal and lateral forces is shown in Figure 5c. Consistent with the measurements obtained by former results [12], no sensitivity to a lateral force i.e., the FBG sensor positioned lateral to the applied load did not experience a Bragg wavelength shift (red curve in Figure 5c), could be detected in the concrete component. Thus, FCSs with two FOSs placed perpendicularly could indeed, in principle, be used to discriminate between transverse and longitudinal forces and to compensate for the temperature.

### 3.2. Performance of Spatial Strain Measurement Using OFDR

**Figure 6:**
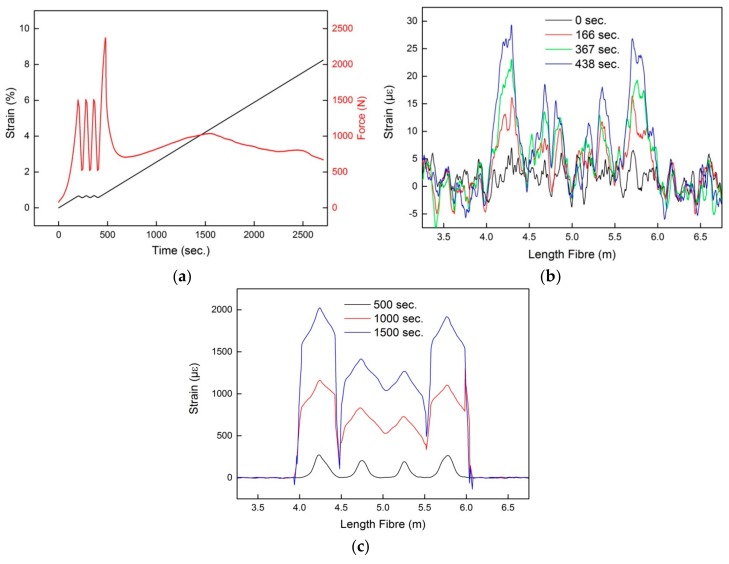
OFDR measurement results; (**a**) Applied strain and force on the concrete block under test; Spatial strain readings of the OFDR before (**b**) and after (**c**) breakage of the concrete block at the load position.

The result of the OFDR measurement is shown in Figure 6. The applied load and force to the concrete block under test is illustrated in Figure 6a. As can be seen, the abrupt force change approximately after 490 s indicates the point of break of the concrete block. From this event on, part of the FCS and thus optical fiber at the position of the crack was not covered in concrete anymore (as indicated in Figure 4c). In Figure 6b the spatial strain readings of the OFDR at different times and hence at different loads are illustrated. Furthermore, the strain readings shown in Figure 6b were performed prior to the breaking of the concrete block. As indicated, four strain peaks were detected using the OFDR. Since the optical fiber used for OFDR was integrated in the FCS in a meander shape (see Figure 1), the FOS of the OFDR experienced four load points. The different magnitudes of the four strain peaks shown in Figure 6b results from non-uniformity of the surface of the concrete block. The spatial strain readings of the OFDR upon breakage of the concrete block at the position of the load are illustrated in Figure 6c. Compared to Figure 6b, the magnitudes of the strain peaks have increased. The increase in amplitude of the strain peaks is due to the higher bend experienced by the concrete block at the position of the breakage. Moreover, the strain peaks broadened in response to increasing load. The broadening of the peaks can be explained as due to strain transfer from the concrete breakage points outwards along the FCS. Therefore, for higher loads the spatial resolution of the OFDR might be limited by the mechanical properties of the FCS.

## 4. Summary

In this communication, the sensor performance of FCS with integrated FOSs under practical conditions has been evaluated. To do so, the FCSs have been embedded in concrete blocks and the sensor performance was evaluated using a 3-point bending test. During embedding in concrete the FCS was closely monitored and only negligible attenuation variation of the light propagating in the optical fiber was detected, which can be attributed to connector tolerances. Moreover, for the evaluation of the FCS, an FBG technique was also applied in addition to the proposed OFDR-based technique. By using the FBG sensors, it could be verified that (i) the response of the FOSs embedded in the FCS has a linear response to applied strain of 0.44 nm/% with a relatively low hysteresis (0.011%) and that (ii) FCSs with two OGFs placed perpendicularly could in principle be used, for example, to discriminate between transverse and longitudinal forces and/or to compensate for temperature of the FOSs, depending on the positon of each OGF sensor relative to the load. Moreover, the OFDR technique verifies that the spatial strain profile of the optical fiber embedded in FCS can be successfully monitored within concrete elements. When performing the 3-point bending test the optical fibers utilized for OFDR measurements were loaded four times and the four load points were illustrated as four strain peaks. Furthermore, the OFDR readings verify that for large loads, the strain propagates from the load points along the FCS and thus causes a broadening of the measured strain peaks. Thus, for large loads the spatial strain resolution of the FCS is limited. Based on the obtained results, currently an appropriate theoretical model is developed in order to model the sensor performance of the FCS embedded in concrete.

## Figures and Tables

**Table 1:** Transmittance before and after applying concrete surrounding FCSs.

Probe:	Performance before Applying Concrete (dB)	Performance after Applying Concrete (dB)
sample #1	57.36	57.4
sample #2	57.02	57.08
sample #3	55.93	55.85
